# Robot-Assisted Sigmoid Colon Conduit for Urinary Diversion: Avoiding Bowel Anastomosis in a Patient with Prior Loop Colostomy and Pelvic Radiation

**DOI:** 10.1590/S1677-5538.IBJU.2026.0133

**Published:** 2026-04-10

**Authors:** Éder Silveira Brazão, Caroline de Almeida Gonçalves, Rodrigo Coelho de Carvalho, Victor Espinheira Santos, Paulo Roberto Stevanato, Stênio de Cássio Zequi

**Affiliations:** 1 AC Camargo Cancer Center Departamento de Urologia São Paulo SP Brasil Departamento de Urologia, AC Camargo Cancer Center, São Paulo, SP, Brasil; 2 AC Camargo Cancer Center Departamento de Cirurgia Colorretal São Paulo SP Brasil Departamento de Cirurgia Colorretal, AC Camargo Cancer Center, São Paulo, SP, Brasil

## Abstract

**Purpose:**

Late radiation-induced genitourinary and gastrointestinal complications after multimodal treatment for pelvic malignancies frequently require complex reconstructive surgery, often necessitating both urinary and fecal diversion (1). In this setting, a colonic segment may provide an effective option for urinary diversion while avoiding bowel anastomosis (2, 3). Minimally invasive robotic approaches have increasingly expanded the feasibility of these complex reconstructions and may reduce morbidity, particularly in frail or previously irradiated patients (4–7). We describe a single-stage robot-assisted sigmoid colon conduit urinary diversion in a patient with a pre-existing loop colostomy.

**Materials and Methods:**

A 46-year-old woman with prior pelvic chemoradiation developed a rectovaginal fistula requiring loop colostomy, followed by bilateral distal ureteral strictures, radiation cystitis, recurrent pyelonephritis, and a large parastomal hernia. After failed conservative management and preoperative nephrostomy placement, a single-stage robot-assisted procedure was performed. The operation included adhesiolysis, parastomal hernia repair, conversion of the loop to a terminal colostomy, and construction of a sigmoid colon urinary conduit. Ureterointestinal anastomoses were completed intracorporeally under indocyanine green fluorescence guidance.

**Results:**

The procedure was completed robotically without intraoperative complications. Total operative time was 95 minutes, with an estimated blood loss of 100 mL. Oral intake resumed on postoperative day 1, and the patient was discharged on day 3. At 6-month follow-up, renal function was preserved, with no recurrent infections or evidence of ureteral obstruction.

**Conclusion:**

Robot-assisted sigmoid colon conduit is a safe and reproducible option for urinary diversion in selected patients requiring concomitant bowel diversion, avoiding bowel anastomosis and reducing reconstructive morbidity.

**Video 1:** Available at: VIDEO

## Data Availability

Data Not Provided
